# 2-Cyano-3-(2,3,6,7-tetra­hydro-1*H*,5*H*-benzo[*ij*]quinolizin-9-yl)prop-2-enoic acid dimethyl sulfoxide monosolvate

**DOI:** 10.1107/S1600536812043383

**Published:** 2012-10-24

**Authors:** Hemant Yennawar, Gang He, Christopher Rumble, Mark Maroncelli

**Affiliations:** a Department of Chemistry, Pennsylvania State University, University Park, PA 16802, USA

## Abstract

In dimethyl sulfoxide solvated 9-(2-carb­oxy-2-cyano­vin­yl)julolidine, C_16_H_16_N_2_O_2_·C_2_H_6_OS, the essentially planar –CH=(CN)–CO_2_H substituent (r.m.s. deviation = 0.014 Å) is almost coplanar with respect to the benzene ring, the dihedral angle between the two planes being 0.48 (2)°. The conformations of the fused, non-aromatic rings were found to be half-chair. In the crystal, the acid molecule forms a hydrogen bond to the O atom of the solvent mol­ecule. The acid mol­ecule is disordered over two positions with respect to the methyl­ene C atoms in a 1:1 ratio. The crystal studied was found to be a racemic twin.

## Related literature

For the synthesis of 9-(2-carb­oxy-2-cyano­vin­yl)julolidine, commonly known as CCVJ, see: Rumble *et al.* (2012 ▶). For a related mol­ecule, see: Liang *et al.* (2009 ▶). For fluorescent-rotor probe studies of CCVJ, see: Sawada *et al.* (1992 ▶); Haidekker *et al.* (2001 ▶). For other applications, see: Iwaki *et al.* (1993 ▶); Haidekker *et al.* (2002 ▶); Tanaka *et al.* (2008 ▶); Hawe *et al.* (2010 ▶); Levitt *et al.* (2011 ▶), Dishari & Hickner (2012 ▶); Howell *et al.* (2012 ▶). For a mechanismic study, see: Rumble *et al.* (2012 ▶). 
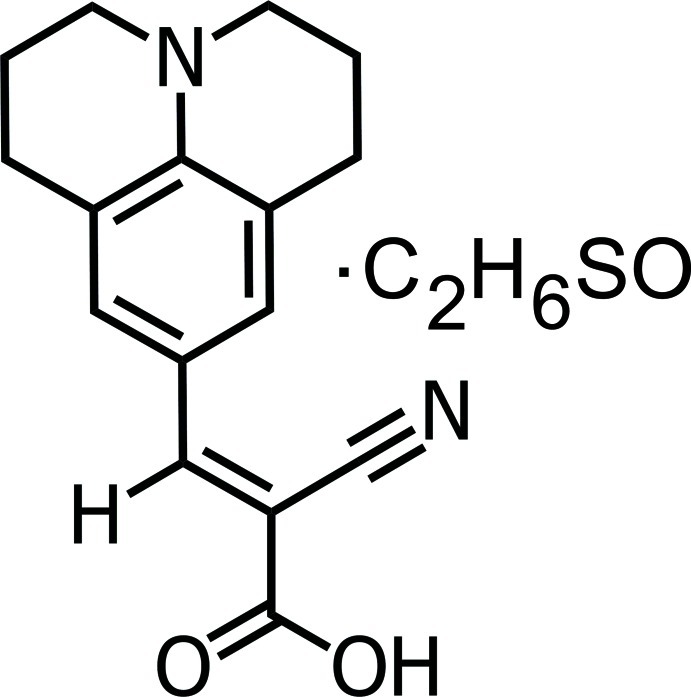


## Experimental

### 

#### Crystal data

C_16_H_16_N_2_O_2_·C_2_H_6_OS
*M* *_r_* = 346.44
Monoclinic, 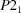

*a* = 10.215 (3) Å
*b* = 7.4588 (19) Å
*c* = 11.819 (3) Å
β = 100.170 (5)°
*V* = 886.4 (4) Å^3^
*Z* = 2
Mo *K*α radiation
μ = 0.20 mm^−1^
*T* = 298 K
0.20 × 0.16 × 0.15 mm

#### Data collection

Bruker SMART APEX CCD area-detector diffractometer
Absorption correction: multi-scan (*SADABS*; Bruker, 2001 ▶) *T*_min_ = 0.961, *T*_max_ = 0.971
5962 measured reflections
3443 independent reflections
2520 reflections with *I* > 2σ(*I*)
*R*_int_ = 0.017

#### Refinement

*R*[*F* ^2^ > 2σ(*F* ^2^)] = 0.051
*wR*(*F* ^2^) = 0.146
*S* = 1.02
3443 reflections
239 parameters
45 restraints
H atoms treated by a mixture of independent and constrained refinement
Δρ_max_ = 0.25 e Å^−3^
Δρ_min_ = −0.23 e Å^−3^
Absolute structure: Flack (1983 ▶), 1096 Friedel pairs
Flack parameter: 0.51 (15)

### 

Data collection: *SMART* (Bruker, 2001 ▶); cell refinement: *SAINT* (Bruker, 2001 ▶); data reduction: *SAINT*; program(s) used to solve structure: *SHELXS97* (Sheldrick, 2008 ▶); program(s) used to refine structure: *SHELXL97* (Sheldrick, 2008 ▶); molecular graphics: *X-SEED* (Barbour, 2001 ▶); software used to prepare material for publication: *SHELXTL* (Sheldrick, 2008 ▶).

## Supplementary Material

Crystal structure: contains datablock(s) I, global. DOI: 10.1107/S1600536812043383/ng5301sup1.cif

Structure factors: contains datablock(s) I. DOI: 10.1107/S1600536812043383/ng5301Isup2.hkl

Supplementary material file. DOI: 10.1107/S1600536812043383/ng5301Isup3.cml

Additional supplementary materials:  crystallographic information; 3D view; checkCIF report

## Figures and Tables

**Table 1:** Hydrogen-bond geometry (Å, °)

*D*—H⋯*A*	*D*—H	H⋯*A*	*D*⋯*A*	*D*—H⋯*A*
O1—H1⋯O3	0.85 (3)	1.83 (3)	2.609 (2)	153 (4)

**Fig. 1.**
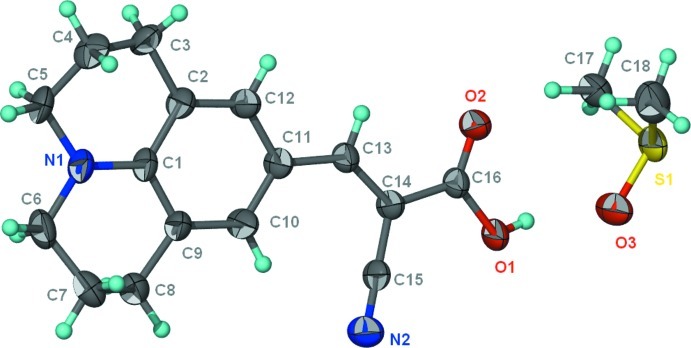
Thermal ellipsoid plot (Barbour, 2001) showing 50% probability displacement ellipsoids; the disorder is not shown.

## supplementary crystallographic information

### Comment

9-(2-carboxy-2-cyanovinyl)julolidine, commonly known as CCVJ, is a fluorescent rotor probe (Sawada *et al.*, 1992; Haidekker *et al.*, 2001) whose fluorescence intensity is strongly modulated by the fluidity of its surroundings. As such, it has been used for studying local fluidity in a variety of contexts (Iwaki *et al.*, 1993; Haidekker *et al.*, 2002; Tanaka *et al.*, 2008; Hawe *et al.*, 2010; Levitt *et al.*, 2011; Dishari & Hickner, 2012; Howell *et al.*, 2012). We have recently studied the rotatory mechanism behind CCVJ's environmental sensitivity, which we showed is an excited-state isomerization (Rumble *et al.*, 2012). Herein we report the crystal structure of CCVJ which we determined in support of this photochemical study.

### Experimental

CCVJ was synthesized by reaction of 9-formyljulolidine and cyanoacetic acid as described by Rumble *et al.* (2012) and purified by silica gel flash chromatography. Orange colored crystals were obtained by slow evaporation of its solution in toluene with a small quantity of DMSO added to increase the solubility, at room temperature.

### Refinement

Hydrogen atoms were placed in calculated positions with C—H 0.93 and 0.97 Å in a riding-model approximation. The acid hydrogen was located in a difference Fourier map and was freely refined.

The acid is disordered over two positions in respect of the five methylene carbons. The occupancy could not be refined so that the disorder was assumed to be a 1:1 type of disorder. Pairs of 1,2-related C–C distances were restrained to within 0.01 Å of each other, and the temperature factors of the primed atoms were set to those of the unprimed ones. Additionally, the anisotropic temperature factors were tightly restrained to be nearly isotropic.

The Flack parameter, refined on 1096 Friedel pairs, was 0.5, which implied that the crystal studied is a racemic twin that crystallizes in a polar space group. The racemic nature also supported the 1:1 type of disorder.

### Crystal data

**Table:**

C_16_H_16_N_2_O_2_·C_2_H_6_OS	*F*(000) = 368
*M**_r_* = 346.44	*D*_x_ = 1.298 Mg m^−^^3^
Monoclinic, *P*2_1_	Mo *K*α radiation, λ = 0.71073 Å
Hall symbol: P 2yb	Cell parameters from 1427 reflections
*a* = 10.215 (3) Å	θ = 2.4–26.3°
*b* = 7.4588 (19) Å	µ = 0.20 mm^−^^1^
*c* = 11.819 (3) Å	*T* = 298 K
β = 100.170 (5)°	Pyramid, orange
*V* = 886.4 (4) Å^3^	0.20 × 0.16 × 0.15 mm
*Z* = 2	

### Data collection

**Table:**

Bruker SMART APEX CCD area-detector diffractometer	3443 independent reflections
Radiation source: fine-focus sealed tube	2520 reflections with *I* > 2σ(*I*)
Graphite monochromator	*R*_int_ = 0.017
φ and ω scans	θ_max_ = 28.3°, θ_min_ = 1.8°
Absorption correction: multi-scan (*SADABS*; Bruker, 2001)	*h* = −10→13
*T*_min_ = 0.961, *T*_max_ = 0.971	*k* = −9→8
5962 measured reflections	*l* = −15→15

### Refinement

**Table:**

Refinement on *F*^2^	Secondary atom site location: difference Fourier map
Least-squares matrix: full	Hydrogen site location: inferred from neighbouring sites
*R*[*F*^2^ > 2σ(*F*^2^)] = 0.051	H atoms treated by a mixture of independent and constrained refinement
*wR*(*F*^2^) = 0.146	*w* = 1/[σ^2^(*F*_o_^2^) + (0.0864*P*)^2^] where *P* = (*F*_o_^2^ + 2*F*_c_^2^)/3
*S* = 1.02	(Δ/σ)_max_ = 0.001
3443 reflections	Δρ_max_ = 0.25 e Å^−^^3^
239 parameters	Δρ_min_ = −0.23 e Å^−^^3^
45 restraints	Absolute structure: Flack (1983), 1096 Friedel pairs
Primary atom site location: structure-invariant direct methods	Flack parameter: 0.51 (15)

### Fractional atomic coordinates and isotropic or equivalent isotropic displacement parameters (Å^2^)

**Table:**

	*x*	*y*	*z*	*U*_iso_*/*U*_eq_	Occ. (<1)
S1	1.57938 (6)	0.5022 (3)	0.08482 (5)	0.0703 (3)	
O1	1.23541 (15)	0.4978 (8)	0.16187 (12)	0.0620 (5)	
H1	1.301 (3)	0.535 (6)	0.134 (3)	0.082 (11)*	
O2	1.37748 (15)	0.5038 (7)	0.32895 (12)	0.0644 (5)	
O3	1.43031 (17)	0.5070 (9)	0.04602 (13)	0.0831 (7)	
N1	0.80423 (18)	0.5001 (7)	0.76083 (14)	0.0546 (5)	
N2	0.9202 (2)	0.5020 (10)	0.19413 (15)	0.0694 (7)	
C1	0.8895 (2)	0.5020 (8)	0.68381 (15)	0.0433 (5)	
C2	1.0286 (2)	0.4971 (8)	0.72132 (16)	0.0483 (5)	
C3	1.0773 (10)	0.478 (2)	0.8505 (5)	0.057 (2)	0.50
H3A	1.1499	0.3929	0.8628	0.069*	0.50
H3B	1.1126	0.5931	0.8802	0.069*	0.50
C4	0.9831 (10)	0.4241 (15)	0.9135 (7)	0.0588 (12)	0.50
H4A	1.0186	0.4433	0.9943	0.071*	0.50
H4B	0.9694	0.2962	0.9024	0.071*	0.50
C5	0.8488 (13)	0.516 (3)	0.8856 (6)	0.061 (2)	0.50
H5A	0.7857	0.4593	0.9265	0.073*	0.50
H5B	0.8568	0.6416	0.9079	0.073*	0.50
C6	0.6611 (6)	0.517 (3)	0.7224 (12)	0.063 (2)	0.50
H6A	0.6365	0.6430	0.7196	0.076*	0.50
H6B	0.6146	0.4578	0.7767	0.076*	0.50
C7	0.6201 (10)	0.4346 (16)	0.6046 (9)	0.0591 (13)	0.50
H7A	0.5245	0.4417	0.5799	0.071*	0.50
H7B	0.6470	0.3099	0.6049	0.071*	0.50
C8	0.6920 (6)	0.5454 (16)	0.5261 (10)	0.0564 (12)	0.50
H8A	0.6774	0.6726	0.5359	0.068*	0.50
H8B	0.6619	0.5138	0.4461	0.068*	0.50
C3'	1.0888 (10)	0.534 (2)	0.8468 (5)	0.057 (2)	0.50
H3'A	1.1476	0.4356	0.8752	0.069*	0.50
H3'B	1.1426	0.6418	0.8502	0.069*	0.50
C4'	0.9974 (9)	0.5554 (15)	0.9189 (6)	0.0588 (12)	0.50
H4'A	0.9893	0.6829	0.9321	0.071*	0.50
H4'B	1.0353	0.5020	0.9922	0.071*	0.50
C5'	0.8576 (13)	0.481 (3)	0.8843 (6)	0.061 (2)	0.50
H5'A	0.8580	0.3550	0.9047	0.073*	0.50
H5'B	0.7990	0.5427	0.9277	0.073*	0.50
C6'	0.6599 (6)	0.488 (3)	0.7296 (11)	0.063 (2)	0.50
H6'A	0.6197	0.5661	0.7792	0.076*	0.50
H6'B	0.6327	0.3658	0.7426	0.076*	0.50
C7'	0.6091 (9)	0.5376 (16)	0.6058 (8)	0.0591 (13)	0.50
H7'A	0.6101	0.6671	0.5983	0.071*	0.50
H7'B	0.5176	0.4977	0.5844	0.071*	0.50
C8'	0.6919 (6)	0.4551 (16)	0.5238 (10)	0.0564 (12)	0.50
H8'A	0.6790	0.3262	0.5207	0.068*	0.50
H8'B	0.6633	0.5028	0.4470	0.068*	0.50
C9	0.8385 (2)	0.4974 (10)	0.56447 (17)	0.0531 (6)	
C10	0.9249 (2)	0.4991 (9)	0.48771 (16)	0.0521 (6)	
H10	0.8901	0.4997	0.4095	0.062*	
C11	1.06346 (19)	0.5000 (9)	0.52212 (15)	0.0432 (5)	
C12	1.1110 (2)	0.4975 (9)	0.64131 (16)	0.0478 (5)	
H12	1.2024	0.4961	0.6671	0.057*	
C13	1.15899 (19)	0.4997 (8)	0.44699 (15)	0.0441 (5)	
H13	1.2469	0.4996	0.4849	0.053*	
C14	1.14494 (19)	0.4994 (8)	0.32993 (15)	0.0422 (5)	
C15	1.0203 (2)	0.4995 (9)	0.25512 (16)	0.0456 (5)	
C16	1.2650 (2)	0.5071 (8)	0.27592 (16)	0.0446 (5)	
C17	1.6110 (5)	0.6841 (7)	0.1863 (4)	0.0650 (14)	
H17A	1.5939	0.7962	0.1465	0.098*	
H17B	1.7022	0.6802	0.2243	0.098*	
H17C	1.5539	0.6728	0.2423	0.098*	
C18	1.6183 (6)	0.3239 (8)	0.1791 (5)	0.0809 (18)	
H18A	1.6065	0.2132	0.1371	0.121*	
H18B	1.5608	0.3257	0.2352	0.121*	
H18C	1.7091	0.3341	0.2172	0.121*	

### Atomic displacement parameters (Å^2^)

**Table:**

	*U*^11^	*U*^22^	*U*^33^	*U*^12^	*U*^13^	*U*^23^
S1	0.0427 (3)	0.1230 (6)	0.0511 (3)	0.0016 (10)	0.0244 (3)	0.0008 (10)
O1	0.0391 (8)	0.1122 (15)	0.0383 (7)	0.008 (3)	0.0162 (6)	0.002 (2)
O2	0.0385 (8)	0.1109 (14)	0.0454 (8)	−0.009 (3)	0.0118 (6)	−0.004 (3)
O3	0.0439 (9)	0.164 (2)	0.0435 (8)	−0.004 (3)	0.0133 (7)	0.016 (3)
N1	0.0482 (10)	0.0773 (13)	0.0434 (9)	−0.007 (3)	0.0220 (8)	−0.010 (3)
N2	0.0437 (11)	0.121 (2)	0.0445 (10)	−0.016 (3)	0.0112 (8)	0.008 (3)
C1	0.0485 (11)	0.0469 (11)	0.0388 (9)	0.004 (3)	0.0192 (8)	0.002 (3)
C2	0.0487 (12)	0.0634 (13)	0.0344 (9)	−0.001 (3)	0.0120 (8)	0.004 (3)
C3	0.056 (2)	0.080 (7)	0.0357 (11)	−0.017 (4)	0.0096 (11)	−0.016 (4)
C4	0.075 (3)	0.072 (3)	0.0313 (13)	−0.003 (4)	0.0137 (14)	0.016 (3)
C5	0.066 (2)	0.082 (6)	0.0405 (11)	0.005 (5)	0.0258 (11)	−0.013 (3)
C6	0.0490 (13)	0.082 (5)	0.0656 (19)	0.009 (4)	0.0303 (11)	0.002 (4)
C7	0.0405 (18)	0.065 (4)	0.0741 (18)	0.005 (4)	0.0173 (14)	0.002 (5)
C8	0.0441 (14)	0.071 (4)	0.0548 (14)	−0.001 (6)	0.0105 (11)	−0.014 (6)
C3'	0.056 (2)	0.080 (7)	0.0357 (11)	−0.017 (4)	0.0096 (11)	−0.016 (4)
C4'	0.075 (3)	0.072 (3)	0.0313 (13)	−0.003 (4)	0.0137 (14)	0.016 (3)
C5'	0.066 (2)	0.082 (6)	0.0405 (11)	0.005 (5)	0.0258 (11)	−0.013 (3)
C6'	0.0490 (13)	0.082 (5)	0.0656 (19)	0.009 (4)	0.0303 (11)	0.002 (4)
C7'	0.0405 (18)	0.065 (4)	0.0741 (18)	0.005 (4)	0.0173 (14)	0.002 (5)
C8'	0.0441 (14)	0.071 (4)	0.0548 (14)	−0.001 (6)	0.0105 (11)	−0.014 (6)
C9	0.0401 (11)	0.0803 (16)	0.0406 (10)	−0.003 (3)	0.0118 (9)	−0.014 (3)
C10	0.0436 (11)	0.0809 (16)	0.0331 (9)	−0.003 (3)	0.0107 (8)	0.016 (3)
C11	0.0419 (11)	0.0534 (12)	0.0368 (9)	−0.005 (3)	0.0134 (8)	0.009 (2)
C12	0.0394 (10)	0.0665 (14)	0.0383 (9)	−0.008 (3)	0.0092 (8)	0.006 (3)
C13	0.0383 (10)	0.0566 (12)	0.0390 (9)	0.005 (3)	0.0110 (8)	0.003 (3)
C14	0.0365 (10)	0.0542 (12)	0.0382 (9)	−0.008 (3)	0.0128 (7)	0.003 (3)
C15	0.0395 (11)	0.0657 (14)	0.0351 (9)	−0.007 (3)	0.0164 (8)	0.004 (3)
C16	0.0391 (11)	0.0596 (14)	0.0373 (9)	0.005 (3)	0.0130 (8)	0.005 (3)
C17	0.041 (3)	0.089 (4)	0.066 (3)	−0.003 (2)	0.013 (2)	−0.006 (2)
C18	0.065 (4)	0.079 (3)	0.103 (4)	−0.011 (3)	0.025 (3)	−0.034 (3)

### Geometric parameters (Å, º)

**Table:**

S1—O3	1.5112 (18)	C3'—C4'	1.380 (7)
S1—C18	1.736 (6)	C3'—H3'A	0.9700
S1—C17	1.802 (5)	C3'—H3'B	0.9700
O1—C16	1.330 (2)	C4'—C5'	1.520 (11)
O1—H1	0.85 (3)	C4'—H4'A	0.9700
O2—C16	1.208 (2)	C4'—H4'B	0.9700
N1—C1	1.366 (2)	C5'—H5'A	0.9700
N1—C6'	1.458 (6)	C5'—H5'B	0.9700
N1—C6	1.458 (6)	C6'—C7'	1.511 (10)
N1—C5	1.470 (6)	C6'—H6'A	0.9700
N1—C5'	1.472 (6)	C6'—H6'B	0.9700
N2—C15	1.142 (3)	C7'—C8'	1.525 (6)
C1—C2	1.412 (3)	C7'—H7'A	0.9700
C1—C9	1.415 (3)	C7'—H7'B	0.9700
C2—C12	1.373 (3)	C8'—C9	1.522 (6)
C2—C3'	1.527 (6)	C8'—H8'A	0.9700
C2—C3	1.527 (6)	C8'—H8'B	0.9700
C3—C4	1.377 (7)	C9—C10	1.374 (3)
C3—H3A	0.9700	C10—C11	1.402 (3)
C3—H3B	0.9700	C10—H10	0.9300
C4—C5	1.518 (11)	C11—C12	1.407 (3)
C4—H4A	0.9700	C11—C13	1.431 (2)
C4—H4B	0.9700	C12—H12	0.9300
C5—H5A	0.9700	C13—C14	1.366 (2)
C5—H5B	0.9700	C13—H13	0.9300
C6—C7	1.513 (10)	C14—C15	1.416 (3)
C6—H6A	0.9700	C14—C16	1.480 (3)
C6—H6B	0.9700	C17—H17A	0.9600
C7—C8	1.525 (7)	C17—H17B	0.9600
C7—H7A	0.9700	C17—H17C	0.9600
C7—H7B	0.9700	C18—H18A	0.9600
C8—C9	1.528 (6)	C18—H18B	0.9600
C8—H8A	0.9700	C18—H18C	0.9600
C8—H8B	0.9700		
			
O3—S1—C18	108.5 (3)	C5'—C4'—H4'A	107.3
O3—S1—C17	103.7 (3)	C3'—C4'—H4'B	107.3
C18—S1—C17	98.95 (13)	C5'—C4'—H4'B	107.3
C16—O1—H1	109 (2)	H4'A—C4'—H4'B	106.9
C1—N1—C6'	124.5 (6)	N1—C5'—C4'	113.4 (10)
C1—N1—C6	120.9 (6)	N1—C5'—H5'A	108.9
C1—N1—C5	123.1 (6)	C4'—C5'—H5'A	108.9
C6'—N1—C5	112.3 (8)	N1—C5'—H5'B	108.9
C6—N1—C5	114.9 (8)	C4'—C5'—H5'B	108.9
C1—N1—C5'	119.6 (6)	H5'A—C5'—H5'B	107.7
C6'—N1—C5'	115.2 (8)	N1—C6'—C7'	112.9 (10)
C6—N1—C5'	119.5 (8)	N1—C6'—H6'A	109.0
N1—C1—C2	120.98 (17)	C7'—C6'—H6'A	109.0
N1—C1—C9	119.88 (19)	N1—C6'—H6'B	109.0
C2—C1—C9	119.02 (16)	C7'—C6'—H6'B	109.0
C12—C2—C1	119.30 (17)	H6'A—C6'—H6'B	107.8
C12—C2—C3'	118.5 (4)	C6'—C7'—C8'	112.4 (12)
C1—C2—C3'	120.7 (4)	C6'—C7'—H7'A	109.1
C12—C2—C3	123.9 (4)	C8'—C7'—H7'A	109.1
C1—C2—C3	116.6 (4)	C6'—C7'—H7'B	109.1
C4—C3—C2	115.6 (7)	C8'—C7'—H7'B	109.1
C4—C3—H3A	108.4	H7'A—C7'—H7'B	107.8
C2—C3—H3A	108.4	C9—C8'—C7'	109.9 (6)
C4—C3—H3B	108.4	C9—C8'—H8'A	109.7
C2—C3—H3B	108.4	C7'—C8'—H8'A	109.7
H3A—C3—H3B	107.4	C9—C8'—H8'B	109.7
C3—C4—C5	116.1 (12)	C7'—C8'—H8'B	109.7
C3—C4—H4A	108.3	H8'A—C8'—H8'B	108.2
C5—C4—H4A	108.3	C10—C9—C1	119.46 (19)
C3—C4—H4B	108.3	C10—C9—C8'	120.3 (5)
C5—C4—H4B	108.3	C1—C9—C8'	119.2 (5)
H4A—C4—H4B	107.4	C10—C9—C8	120.8 (5)
N1—C5—C4	106.8 (9)	C1—C9—C8	117.3 (5)
N1—C5—H5A	110.4	C9—C10—C11	122.84 (17)
C4—C5—H5A	110.4	C9—C10—H10	118.6
N1—C5—H5B	110.4	C11—C10—H10	118.6
C4—C5—H5B	110.4	C10—C11—C12	116.29 (16)
H5A—C5—H5B	108.6	C10—C11—C13	125.74 (17)
N1—C6—C7	110.5 (10)	C12—C11—C13	117.96 (18)
N1—C6—H6A	109.5	C2—C12—C11	122.99 (19)
C7—C6—H6A	109.5	C2—C12—H12	118.5
N1—C6—H6B	109.5	C11—C12—H12	118.5
C7—C6—H6B	109.5	C14—C13—C11	131.90 (19)
H6A—C6—H6B	108.1	C14—C13—H13	114.1
C6—C7—C8	104.7 (13)	C11—C13—H13	114.1
C6—C7—H7A	110.8	C13—C14—C15	123.68 (17)
C8—C7—H7A	110.8	C13—C14—C16	119.35 (18)
C6—C7—H7B	110.8	C15—C14—C16	116.93 (16)
C8—C7—H7B	110.8	N2—C15—C14	179.0 (6)
H7A—C7—H7B	108.9	O2—C16—O1	123.34 (18)
C7—C8—C9	104.1 (7)	O2—C16—C14	124.06 (17)
C7—C8—H8A	110.9	O1—C16—C14	112.20 (18)
C9—C8—H8A	110.9	S1—C17—H17A	109.5
C7—C8—H8B	110.9	S1—C17—H17B	109.5
C9—C8—H8B	110.9	H17A—C17—H17B	109.5
H8A—C8—H8B	108.9	S1—C17—H17C	109.5
C4'—C3'—C2	114.8 (7)	H17A—C17—H17C	109.5
C4'—C3'—H3'A	108.6	H17B—C17—H17C	109.5
C2—C3'—H3'A	108.6	S1—C18—H18A	109.5
C4'—C3'—H3'B	108.6	S1—C18—H18B	109.5
C2—C3'—H3'B	108.6	H18A—C18—H18B	109.5
H3'A—C3'—H3'B	107.5	S1—C18—H18C	109.5
C3'—C4'—C5'	119.9 (9)	H18A—C18—H18C	109.5
C3'—C4'—H4'A	107.3	H18B—C18—H18C	109.5
			
C6'—N1—C1—C2	−173.9 (13)	C3'—C4'—C5'—N1	−38 (2)
C6—N1—C1—C2	175.9 (12)	C1—N1—C6'—C7'	−19 (2)
C5—N1—C1—C2	8.1 (14)	C6—N1—C6'—C7'	51 (6)
C5'—N1—C1—C2	−3.9 (13)	C5—N1—C6'—C7'	159.2 (15)
C6'—N1—C1—C9	2.1 (15)	C5'—N1—C6'—C7'	170.7 (15)
C6—N1—C1—C9	−8.2 (15)	N1—C6'—C7'—C8'	44 (2)
C5—N1—C1—C9	−175.9 (12)	C6'—C7'—C8'—C9	−51.9 (15)
C5'—N1—C1—C9	172.0 (11)	N1—C1—C9—C10	−179.9 (6)
N1—C1—C2—C12	179.4 (6)	C2—C1—C9—C10	−3.9 (10)
C9—C1—C2—C12	3.4 (9)	N1—C1—C9—C8'	−11.4 (11)
N1—C1—C2—C3'	−14.6 (11)	C2—C1—C9—C8'	164.6 (7)
C9—C1—C2—C3'	169.4 (9)	N1—C1—C9—C8	17.6 (10)
N1—C1—C2—C3	3.6 (11)	C2—C1—C9—C8	−166.4 (7)
C9—C1—C2—C3	−172.4 (9)	C7'—C8'—C9—C10	−155.4 (9)
C12—C2—C3—C4	−160.1 (10)	C7'—C8'—C9—C1	36.2 (13)
C1—C2—C3—C4	15.5 (16)	C7'—C8'—C9—C8	−56.6 (13)
C3'—C2—C3—C4	124 (4)	C7—C8—C9—C10	149.4 (9)
C2—C3—C4—C5	−45.1 (18)	C7—C8—C9—C1	−48.3 (11)
C1—N1—C5—C4	−33.8 (19)	C7—C8—C9—C8'	52.9 (15)
C6'—N1—C5—C4	148.0 (15)	C1—C9—C10—C11	1.9 (10)
C6—N1—C5—C4	157.8 (14)	C8'—C9—C10—C11	−166.4 (8)
C5'—N1—C5—C4	40 (5)	C8—C9—C10—C11	163.8 (7)
C3—C4—C5—N1	52.5 (19)	C9—C10—C11—C12	0.5 (9)
C1—N1—C6—C7	32 (2)	C9—C10—C11—C13	179.2 (7)
C6'—N1—C6—C7	−84 (7)	C1—C2—C12—C11	−0.9 (9)
C5—N1—C6—C7	−159.6 (14)	C3'—C2—C12—C11	−167.3 (9)
C5'—N1—C6—C7	−148.5 (14)	C3—C2—C12—C11	174.6 (9)
N1—C6—C7—C8	−62.6 (16)	C10—C11—C12—C2	−1.0 (9)
C6—C7—C8—C9	68.9 (11)	C13—C11—C12—C2	−179.8 (6)
C12—C2—C3'—C4'	171.6 (9)	C10—C11—C13—C14	0.4 (10)
C1—C2—C3'—C4'	5.5 (15)	C12—C11—C13—C14	179.1 (6)
C3—C2—C3'—C4'	−75 (3)	C11—C13—C14—C15	0.0 (10)
C2—C3'—C4'—C5'	20.9 (17)	C11—C13—C14—C16	177.4 (7)
C1—N1—C5'—C4'	28.6 (19)	C13—C14—C16—O2	4.2 (10)
C6'—N1—C5'—C4'	−160.5 (15)	C15—C14—C16—O2	−178.3 (6)
C6—N1—C5'—C4'	−151.2 (15)	C13—C14—C16—O1	177.2 (6)
C5—N1—C5'—C4'	−83 (7)	C15—C14—C16—O1	−5.3 (8)

### Hydrogen-bond geometry (Å, º)

**Table:**

*D*—H···*A*	*D*—H	H···*A*	*D*···*A*	*D*—H···*A*
O1—H1···O3	0.85 (3)	1.83 (3)	2.609 (2)	153 (4)
